# First Insight into the Diversity and Antibacterial Potential of Psychrophilic and Psychotrophic Microbial Communities of Abandoned Amber Quarry

**DOI:** 10.3390/microorganisms9071521

**Published:** 2021-07-16

**Authors:** Margarita Shevchenko, Stanislav Sukhikh, Olga Babich, Svetlana Noskova, Svetlana Ivanova, Valery Lisun, Viktoriya Skripskaya, Andrey Lomtev, Maria Zimina

**Affiliations:** 1 Institute of Living Systems, Immanuel Kant Baltic Federal University, 236016 Kaliningrad, Russia; lionsorciere@gmail.com (M.S.); stas-asp@mail.ru (S.S.); olich.43@mail.ru (O.B.); svykrum@mail.ru (S.N.); lisun.valer@yandex.ru (V.L.); visknot@gmail.com (V.S.); divepro39@mail.ru (A.L.); mariia.zimina@list.ru (M.Z.); 2 Natural Nutraceutical Biotesting Laboratory, Kemerovo State University, 650043 Kemerovo, Russia; 3 Department of General Mathematics and Informatics, Kemerovo State University, 650043 Kemerovo, Russia

**Keywords:** bacteriocins, extremophiles (psychrophiles), amber quarry, cellulolytics, rarefaction curves

## Abstract

Natural habitats, including extreme ones, are potential sources of new antimicrobial compound producers, such as bacteriocins and enzymes, capable of degrading the matrix polysaccharides of bacterial biofilms. This study aimed to investigate biodiversity and evaluate the antibacterial potential of psychrophilic and psychrotrophic microbial communities of the flooded Walter amber quarry (Kaliningrad region, Russia). As a result of 16S rDNA high-throughput profiling, 127 genera of bacteria belonging to 12 phyla of bacteria were found in sediment samples: *Acidobacteria* sp., *Actinobacteria* sp., *Armatimonadetes* sp., *Bacteroidetes* sp., *Chloroflexi* sp., *Cyanobacteria* sp., *Firmicutes* sp., *Gemmatimonadetes* sp., *Planctomycetes* sp., *Proteobacteria* sp., *Tenericutes* sp., and *Verrucomicrobia* sp. The dominant bacteria groups were the families Ruminococcaceae and Lachnospiraceae, belonging to the order Clostridiales phylum *Firmicutes*. Analysis of enrichment cultures obtained from sediments showed the presence of antibacterial and cellulolytic activity. It seems likely that the bacteria of the studied communities are producers of antimicrobial compounds and have the potential for biotechnological use.

## 1. Introduction

Antibiotic therapy is the most significant scientific achievement of the twentieth century in terms of its impact on human morbidity and mortality, but today, there are some problems that limit the use of antibiotics. The emergence of antibiotic-resistant pathogens is the main problem, while the high costs and risks associated with the development of new products lead to a shortage of new families of antibiotics that could compensate for resistance to existing ones [1,2]. Administration of broad-spectrum antibiotics can lead to collateral damage to the commensal microbiota, which plays an essential role in the health of the host and the growth in the number of atopic and autoimmune diseases [3,4,5].

Alternatives to antibiotics studied to date include plant compounds, bacteriophages, phage lysines, RNA therapies, probiotics, antimicrobial peptides, and enzymes. Bacteriocins, which are small ribosomally synthesized peptides produced by bacteria, have high activity against many clinical targets and have mechanisms of action different from antibiotics [6,7,8,9,10,11]. To date, several bacteriocins with a broad or a narrow spectrum activity are known, which can be applied to combat infections of unknown etiology and control target pathogens without adversely affecting commensal populations [12,13,14]. There is also information about the successful use of protein cocktails that include bacterial cellulases exhibiting enzymatic activity against the matrix polysaccharides of antibiotic-resistant biofilms [15].

Extremophilic microbial communities are an interesting source of potential producers of new antimicrobial compounds. Molecular and physiological adaptations of extremophiles, including the high activity and structural flexibility of peptides and enzymes, allow the latter to be used in molecular biology, waste processing, paper and food industries, pharmacology, bioremediation, and medicine, while understanding the adaptive mechanisms and structures of communities can help to develop strategies for obtaining compounds that can later be used for medical, scientific, and commercial purposes [16,17,18]. 

This study aimed to investigate biodiversity and evaluate the antibacterial potential of psychrophilic and psychrotrophic microbial communities of the flooded amber quarry (Walter, Kaliningrad region, Russia).

## 2. Materials and Methods

### 2.1. Sample Collection

**Figure 1:**
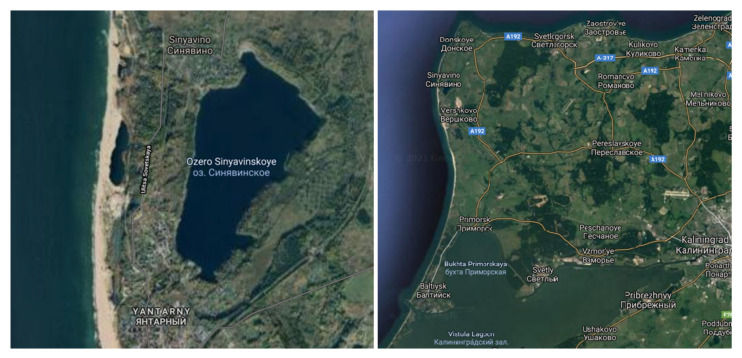
Satellite map of Walter quarry, Kaliningrad region, Russia.

The samples were collected from the disused and flooded Walter amber quarry (Figure 1), located near the village of Yantarny (Kaliningrad region, Russia) (54°53′04.4′′ N, 19°56′53.2′′ E). A feature of the quarry is the presence of a thermocline at a depth of 10 m. The temperature difference is about 10 °C at a reservoir depth of 30 m. The temperature at a depth of 10–30 m is stable throughout the year and is about 4–7 °C. 

The cold-water layer formed by spring waters is a potential habitat for psychrophilic and psychrotrophic bacteria. The flooded forest, building structures, and blue clays in the bottom sediments of the quarry, along with the low water temperature, create conditions for the functioning of complex, including cellulolytic and microbial communities.

Sampling was carried out in the spring of 2020. Sediments are a mixture of sand and blue clay. Sediments were sampled at a depth of 10–25 m a 5 cm into the sediment layer. Samples were collected in 50 mL sterile plastic tubes and transported in a portable refrigerator at the 4 °C.

### 2.2. DNA Isolation and Purification

Total DNA from sediment samples was isolated by phenol-chloroform extraction. 5 g sediments aliquots were preliminarily washed with 120 mM potassium phosphate buffer solution (K_2_HPO_4_/KH_2_PO_4_) by vortexing for 5 min. The resulting suspensions were supplemented with 10 mL of lysis buffer (100 mM Tris-HCl pH 8.0, 100 mM EDTA, 15 mM NaCl, 2 μg/mL lysozyme), after which the samples were incubated for 30 min at 37 °C. After the specified time, the suspensions were subjected to three freeze-thaw cycles at −20 °C and 50 °C, respectively. Next, in the final solution, up to 2% sodium dodecyl sulfate (SDS) and proteinase K (5 μg/mL) were added. The suspensions were incubated for 16 h at room temperature using a rotary shaker for constant stirring. The lysed suspension was centrifuged for 10 min at 10,000× *g*. The resulting supernatant was gently stirred until the formation of an emulsion with 0.5 volume (V) of chilled chloroform and 0.5 V of chilled phenol for deproteinization. The emulsion was centrifuged for 10 min at 10,000× *g*. The resulting aqueous phase was transferred to a new tube, after which the procedure was repeated 3 times with the addition of 1 V chilled chloroform. The DNA contained in the aqueous phase was precipitated using 1/10 V 3M NaAc pH 5.2 and 2 V chilled 96% ethanol. The precipitated DNA was washed with ethanol, dried at room temperature (RT), and dissolved in 50 μL of mQ water. The total DNA concentration was measured using a Qubit 2.0 fluorometer (Thermo Fisher Scientific, Waltham, MA, USA).

### 2.3. 16S rRNA Gene Amplicon Sequencing

Libraries of the V4 region of the 16S rRNA gene for high throughput sequencing (NGS) were obtained using the double-indexed primer system described by Fadrosh et al. [19] to obtain information about the structure of the community. The primer annealing regions corresponded to primers F515 5′-GTGBCAGCMGCCGCGGTAA-3′ and R806 5′-GGACTACHVGGGTWTCTAAT-3′. The samples were amplified using the qPCRmix-HS SYBR reaction mixture (Eurogen, Moscow, Russia) in duplicate. Amplification was performed using a CFX96 real-time PCR system (BioRad, Hercules, CA, USA). Libraries were purified using the Cleanup Mini kit (Eurogen, Moscow, Russia). The quality of the purified libraries was checked by agarose gel electrophoresis. The library concentrations were measured using a Qubit 2.0 fluorometer (Thermo Fisher Scientific, Waltham, MA, USA). The purified libraries were pooled in an equimolar ratio. The final pool concentration was 13 pmol/μL. Pairwise end sequencing was performed in the MiSeq system using the MiSeq v2 reagent kit (500 cycles) (Illumina Inc., San Diego, CA, USA).

### 2.4. Data Processing

Read processing was performed using scripts from Fadrosh et al. [19] and included removing primer sequences, filtering of reads using the Phred algorithm, and assembly of paired-end reads using the SeqPrep program (https://github.com/jstjohn/SeqPrep (accessed on 20 February 2021). Demultiplexing and taxonomic analysis was carried out using the QIIME 2 software package [20]. The quality of the sequences was checked using the q2-dada2 Qiime2 plugin. The resulting operational taxonomic units (OTU) were analyzed using the q2-diversity Qiime2 plugin to calculate alpha diversity. Next, the taxonomic composition of the samples was studied using a naive Bayes classifier via the SILVA database and the q2-feature-classifier plugin [21]. Belonging to a specific family and genus was determined based on the OTU tables. Hydrolytic enzymes were predicted from the 16S rDNA sequence information using the UniProt database [22].

The Shannon and Chao1 indexes were calculated to assess alpha diversity or species diversity within a community. The Shannon index shows the diversity of the community in terms of the frequency of occurrence of taxa (the higher the index, the more diverse the community is considered). The Chao1 index measures diversity considering the occurrence of species (the less a given species occurs in a sample, the more weight it has). To determine the effect of reading depth (the number of readings in each sample) on alpha diversity, rarefaction curves were constructed showing the number of species detected at a given number of reads [23,24]. 

### 2.5. Testing of Antibacterial and Cellulolytic Activity 

Enrichment cultures were obtained from sediment samples to assess the possible antibacterial activity. In total, 5 g of sediments were placed in 100 mL of liquid modified Brunner mineral medium (Table 1) and cultured at 17 °C in a shaker thermostat for two weeks.

Antimicrobial activity was evaluated using the disk diffusion method. 50 mL of enrichment cultures were centrifuged for 20 min at 5000 rpm. The resulting supernatant was filtered through a 0.22 µm vacuum filter (Millipore, Burlington, MA, USA) into sterile 50 mL tubes. 

Model strains of *E. coli* and *B. subtilis* were used as test cultures to evaluate the antimicrobial activity. Inoculums of test bacteria were obtained by transferring 10 colonies of a daily culture from a petri dish into a liquid LB culture medium. The cultures were incubated in a shaker incubator at 25 °C up to OD_600_ equal to 0.10–0.15. Then, 500 μL of inoculums were applied to a petri dish with LB agar (agar—1.5%) and spread with a Drigalski spatula until completely dry. Sterile paper disks were soaked in the filtered supernatant of the studied enrichment cultures and placed on the test culture plates. Sterile media discs were used as a negative control. Petri dishes with inocula were incubated for 24 h at a temperature of 20 °C. Antimicrobial activity was evaluated by the presence of a lysis zone around the paper disk. 

To determine the ability of the studied microbial communities to degrade cellulose, enrichment cultures were obtained from sediment samples on various liquid nutrient media (Table 2) using carbomethylcellulose and amorphous cellulose as the only carbon sources.

The enrichment cultures were grown for one and a half months at 17 °C, followed by passaging on agar nutrient media. For passage 1 mL 1 week-grown cell suspension was inoculated into 20 mL fresh medium, followed by passaging on agar nutrient media. The hydrolytic activity of bacteria was determined by their ability to discolor the medium [25,26].

The bacterial strains from enrichment cultures were isolated from the forming colonies and plated on solid nutrient media to determine the cellulolytic activity. Petri dishes with newly formed colonies were filled with 0.1% Congo red solution, left for 15 min, and then treated with 1 M sodium chloride solution. Colonies showing dye discoloration were taken as positive cellulose-degrading bacteria [27]. 

## 3. Results

### 3.1. Microbial Diversity

16S rDNA profiling of microbial communities of sediment samples obtained from depths of 10 (sample V1), 15 (sample V3), 17 (sample V4), and 20 (sample V5) meters was performed. The total number of reads for each sample exceeds 5000. 

**Figure 2:**
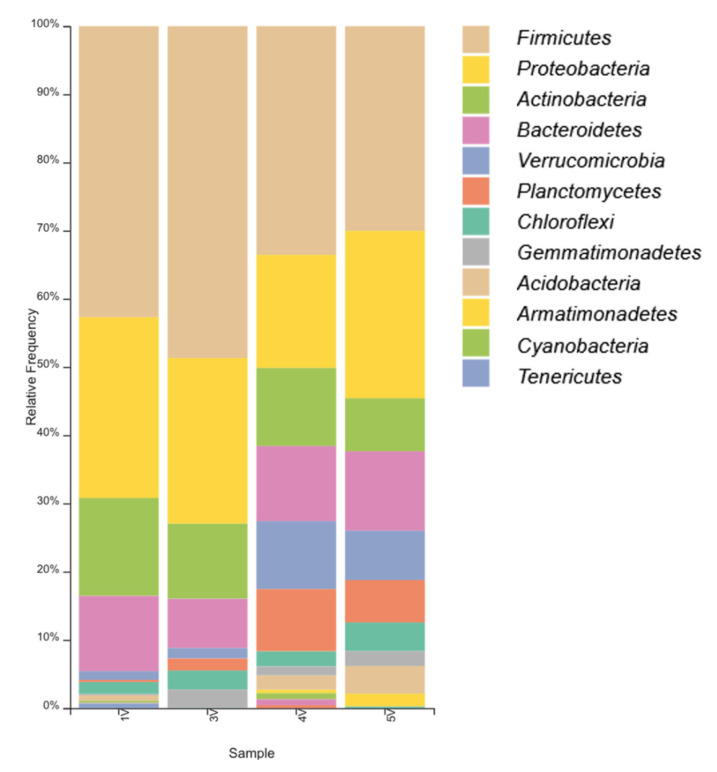
Phyla of bacteria found in sediment samples.

When analyzing the sequencing data, a total of 127 genera belonging to 12 phyla of bacteria were found: *Acidobacteria* sp., *Actinobacteria* sp., *Armatimonadetes* sp., *Bacteroidetes* sp., *Chloroflexi* sp., *Cyanobacteria* sp., *Firmicutes* sp., *Gemmatimonadetes* sp., *Planctomycetes* sp., *Proteobacteria* sp., *Tenericutes* sp., *and Verrucomicrobia* sp. (Figure 2). 

In sediment samples V1 (Appendix A), 40 families of bacteria were found, of which 41% belong to the families Ruminococcaceae, Lachnospiraceae, and Christensenellaceae belonging to the order Clostridiales of the *Firmicutes* phylum. The next most abundant phyla are the *Proteobacteria* (23% of all detected bacteria), half of which belong to the Burkholderiaceae family of the *Gammaproteobacteria* class. 

In addition, 4–5% of the entire community was attributed to the families Geodermatophilaceae of the *Actinobacteria* phylum and Bacteroidaceae of the *Bacteroidetes* phylum. 

In sediment samples V3, families Ruminococcaceae and Lachnospiraceae were also the most numerous and accounted for 31–37% of the total bacteria. However, in this sample, 9% of bacteria belonged to the family Bacillaceae, order Bacillales and 5% *Rhizobiaceae*, *Proteobacteria* phylum (Appendix B). 

In samples V4 (Appendix C), 12% of the total number of bacteria belonged to the family Bifidobacteriaceae and 10% to the *Planctomycetes* phylum. Also, 4% of the community belonged to the family Opitutaceae. 

In samples V5 (Appendix D), 8% of the bacteria detected belonged to the family Moraxellaceae and 3% to Mycobacteriaceae. 

The maximum values of Shannon indexes for samples V1, V3, V4, and V5 are, respectively, 6.79, 6.83, 6.37, 6.31, and Chao1 indexes—128, 133, 104, and 100. The close values are explained by the belonging of the samples to the same microbial community. The rarefaction curves show that the obtained number of reads is sufficient to determine the species diversity in the samples.

Extremophilic (psychrophilic) bacteria are bacteria that can survive at low temperatures (below −10 °C). Psychrophiles also function at temperatures above 20 °C [4]. The bacteria described in our study were cultivated at 17 °C to cover the widest range of psychrophilic and psychrotrophic microorganisms.

### 3.2. Antibacterial and Cellulolytic Activity

**Figure 3:**
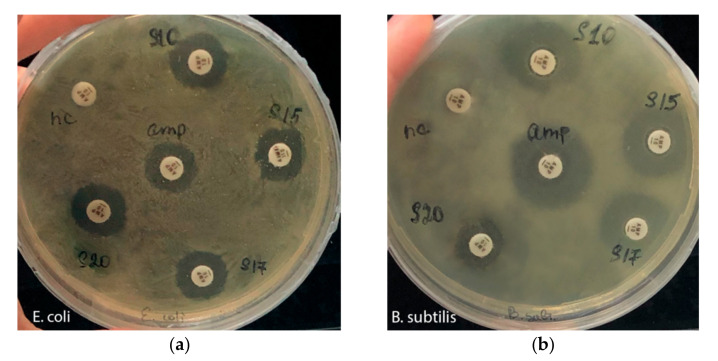
The evaluation of the antimicrobial activity of enrichment cultures against (**a**) *E. coli* and (**b**) *B. subtilis*: S10–S20—enrichment cultures, nc—negative control; amp—positive control (ampicillin).

To evaluate the antibacterial activity, 4 enrichment cultures were obtained from sediment samples collected from the flooded Walter amber quarry, corresponding to sampling depths of 10 m (S10), 15 m (S15), 17 m (S17), and 20 m (S20). The evaluation of antimicrobial activity showed that all four enrichment cultures are active against test bacteria *E. coli* and *B. subtilis* (Table 3, Figure 3) and can include the phyla of psychrophilic and psychrotrophic bacteria producing antimicrobial compounds.

Analysis of the results of 16S rDNA profiling according to the UniProt database showed that cellulolytic enzymes are potentially synthesized in 67 genera of bacteria that are part of the studied microbial communities, including the genera *Paenibacillus*, *Bacteroides*, *Streptococcus*, *Bacillus*, *Lactobacillus*, *Mycobacterium*, *Roseburia*, *Flavobacterium*, *Cellulomonas*, *Cellvibrio*, and *Lachnoclostridium*. 

**Figure 4:**
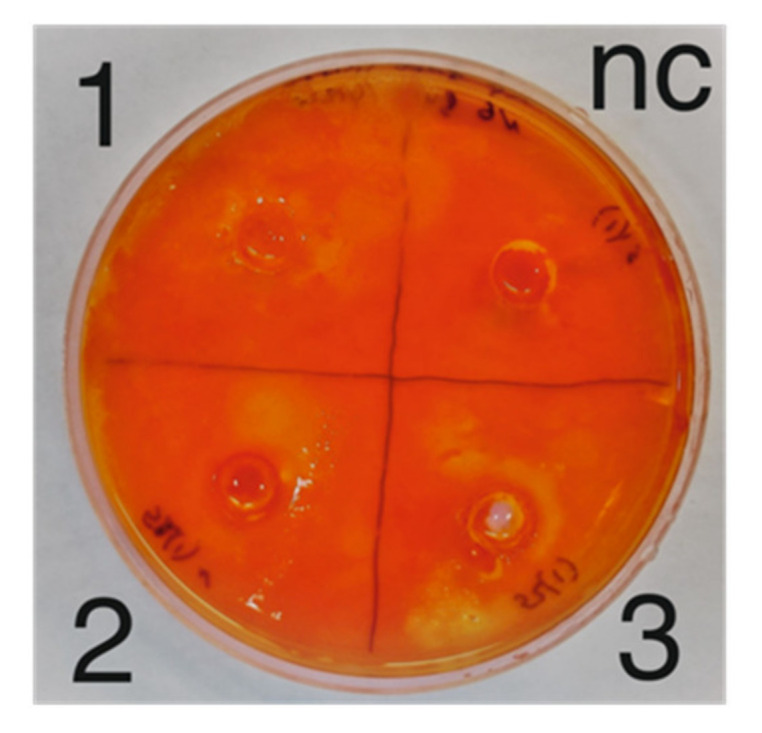
The evaluation of the cellulolytic activity of bacterial strains isolated from enrichment cultures: 1, 2, 3-cellulose-degrading bacterial strains, nc-negative control.

As a result of growing enrichment cultures of cellulolytic bacteria, microbial growth was shown on nutrient media 3, 4 and 5 (Table 2). The analysis of hydrolytic activity performed using the method Congo red has demonstrated the lack of activity of bacterial strains isolated from enrichment cultures that were grown on medium 4. At the same time, enrichment cultures grown on nutrient media 3 and 5 showed a high ability to cellulose degradation. In total, 3 cellulose-degrading bacterial strains were isolated from these enrichment cultures, and their hydrolytic activity was demonstrated (Figure 4). The taxonomic affiliation of the isolated strains remains to be determined. 

## 4. Discussion

Cold habitats occupy about three-quarters of the Earth’s surface [28]. Such habitats are represented by deep sea, permafrost, glaciers, cold-water lakes, soils, deserts, and caves [29] and are successfully colonized by various communities of psychrophilic and psychrotrophic prokaryotic and eukaryotic organisms capable of maintaining high metabolic activity in low-temperature conditions [17].

Today, psychrophilic microbial communities occupying ecological niches with extremely low temperatures are of the greatest interest due to their biotechnological potential. Representatives of these consortia and their metabolic products are successfully used in various industries, including food production, waste processing, mining, environmental bioremediation, agriculture, medicine, and molecular diagnostics [30].

The study of psychrophilic bacteria as new tools in pharmaceuticals is especially promising. At the moment, several promising psychrophilic strains have been discovered that are producers of new highly active antimicrobial compounds [31], such as antarticin-NF3, synthesized by the Antarctic bacterium *Pseudoalteromonas* [32], and the bacteriocins of *Pseudomonas antarctica* PAMC 27494 [33]. 

In addition, among psychrophilic and psychrotrophic bacteria, there are often producers of enzymes that mediate the degradation of insoluble natural biopolymers, such as cellulose and chitin. Currently, the possibility of using these enzymes as antimicrobial agents in a complex therapy directed against pathogenic bacteria that form biofilms resistant to antibiotics is actively studied [34,35]. 

Perchrophils and psychrotrophs are phylogenetically diverse groups and include various families of bacteria and archaea [31]. This study showed that the microbial communities of the Walter quarry are represented by the families Ruminococcacea, Lachnospiraceae, Burkholderiaceae, Bacteroidaceae, Geodermatophilaceae, Bacillaceae, Rhizobiaceae, Planctomycetes, Opitutaceae, Moraxellaceae, and Mycobacteriaceae. 

Representatives of the Ruminococcaceae are morphologically diverse and include bacilli, cocci, and pleomorphic forms. Several species of them are free-living bacteria capable of decomposing cellulose [36,37]. The Lachnospiraceae are anaerobic chemoorganotrophs with various hydrolytic enzymes capable of hydrolyzing xylan, galactose, starch, and cellulose [38,39]. Representatives of the Geodermatophilaceae have been isolated from habitats with different climatic conditions, including deserts, rocks, soil, surfaces of rocks and monuments. All of these groups of microorganisms are producers of bacteriocins [40].

The Bacillaceae consists mainly of aerobic or microaerophilic chemoorganotrophic gram-positive bacteria that form endospores. They are widely represented in natural communities and are confirmed producers of bacteriocins [41] as well as representatives of the Rhizobiaceae [42]. 

The uncultured bacteria of the *Planctomycetes* phylum in the sediment are also interesting as potential producers of new bacteriocins [43].

The Opitutaceae are Gram-negative bacteria present in soils, hot springs, lakes, peat bogs, and inside the digestive tract of invertebrates and are capable of hydrolyzing cellulose [44]. 

The evaluation of antibacterial (Figure 4) and cellulolytic activity carried out in this study confirm the presence of potential producers of new antimicrobial compounds in the sediment communities of the Walter quarry.

## 5. Conclusions

The study of the biodiversity of psychrophilic and psychrotrophic microbial communities of the flooded Walter quarry (Kaliningrad region, Russia) revealed that the dominant groups of bacteria are the Ruminococcaceae and Lachnospiraceae families, belonging to the order Clostridiales, *Firmicutes* phylum. Bacteria of these families, as well as the *Proteobacteria, Acidobacteria, Actinobacteria* phylum and other minor groups, represented to a lesser extent, are potential producers of antimicrobial compounds and have a high potential for use in biotechnology.

## Figures and Tables

**Table 1:** The composition of the modified Brunner mineral medium.

Component	Content, g/L
MgSO_4_ × 7H_2_O	0.2
CaCl_2_ × 2H_2_O	0.02
KH_2_PO_4_	1.0
K_2_HPO_4_	1.0
(NH_4_)2SO_4_	0.5
FeCl_3_	0.02
Peptone/casamino acids	10.0
Yeast extract	5.0

**Table 2:** The composition of specialized nutrient medium for cellulolytic bacterial cultures.

Components	Mediums				
	1	2	3	4	5
NaCl, g/L	20	15	10	-	-
KCl, g/L	2.0	2.0	2.0	0.5	0.5
NH_4_Cl, g/L	0.25	0.25	0.25	-	-
K_2_HPO_4_, g/L	2.5	2.5	2.5	1.8	1.8
MgSO_4_, g/L	-	-	-	0.9	0.9
NaNO_3_, g/L	-	-	-	1	1
Carbomethylcellulose, g/L	-	-	-	5	2
Amorphous cellulose, g/L	5	5	5	-	-

**Table 3:** The evaluation of the antimicrobial activity of enrichment cultures against *E. coli* and *B. subtilis*.

Sample	Lysis Zone Diameter, mm	
	*E. coli*	*B. subtilis*
S10	16	18
S15	13	17
S17	14	17
S20	15	13
Ampicillin	13	21
Negative control	-	-

**Figure A1:**
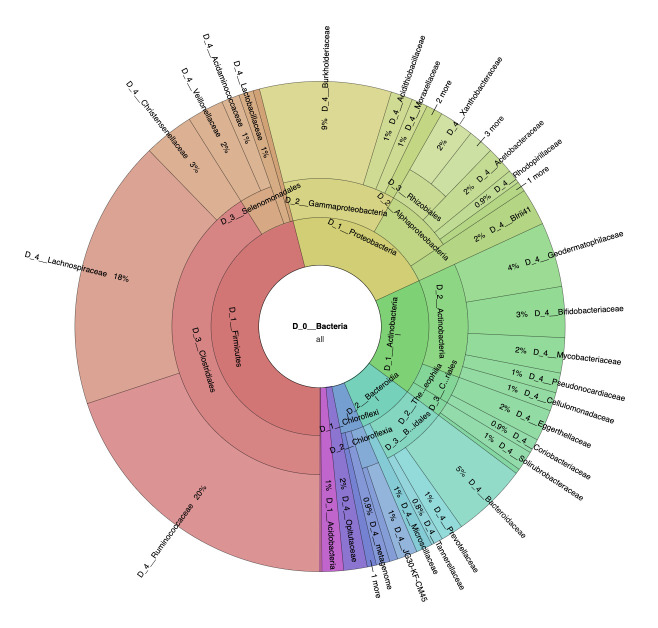
The percentage of bacterial families found in the V1 sample.

**Figure A2:**
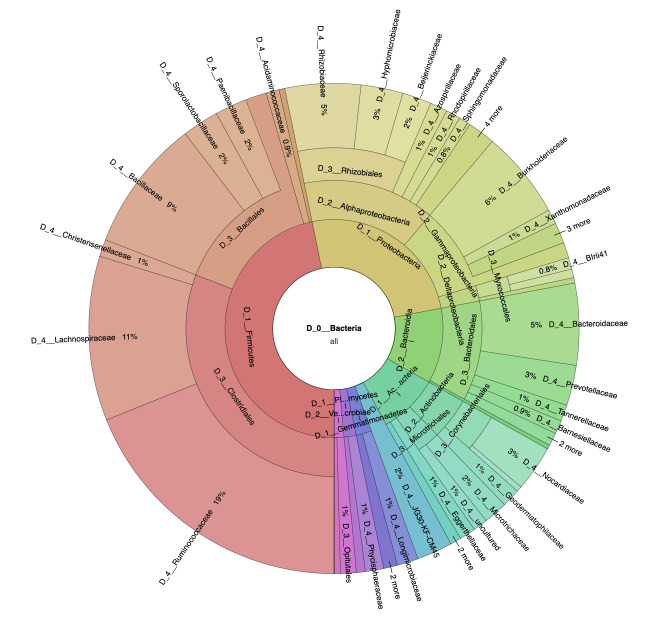
The percentage of bacterial families found in the V3 sample.

**Figure A3:**
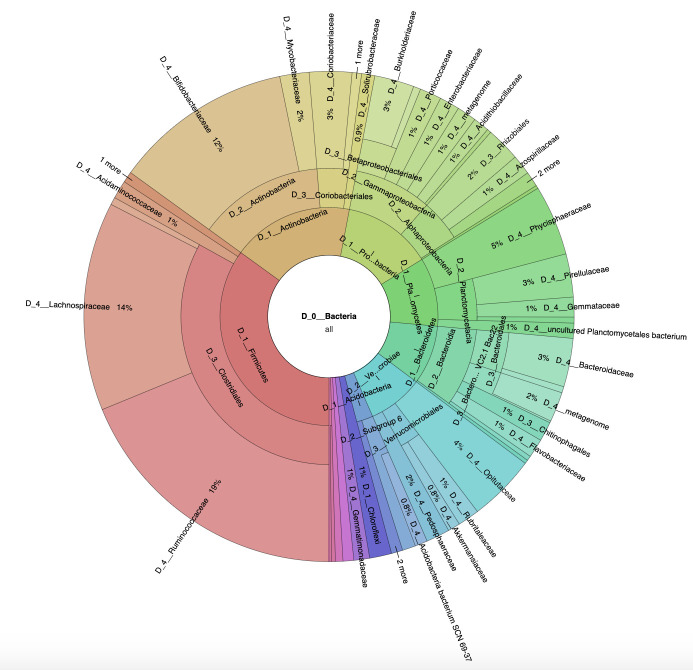
The percentage of bacterial families found in the V4 sample.

**Figure A4:**
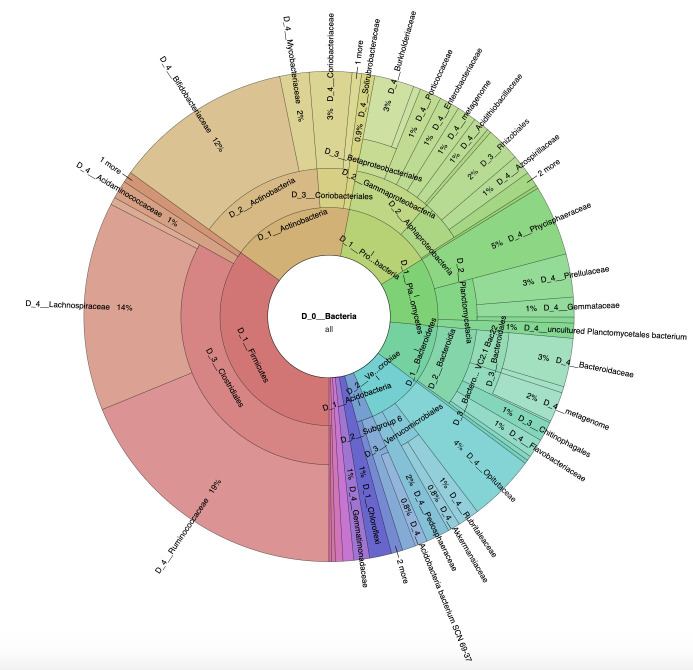
The percentage of bacterial families found in the V5 sample.

## Data Availability

Data are contained within the article.
